# Adaptation-Induced Compression of Event Time Occurs Only for Translational Motion

**DOI:** 10.1038/srep23341

**Published:** 2016-03-22

**Authors:** Michele Fornaciai, Roberto Arrighi, David C. Burr

**Affiliations:** 1 Department of Neuroscience, Psychology, Pharmacology and Child health (NEUROFARBA), University of Florence, via di San Salvi 12, Firenze 50139, Italy; 2 Institute of Neuroscience, National Research Council (CNR), Viale Moruzzi 1, Pisa 56124, Italy

## Abstract

Adaptation to fast motion reduces the perceived duration of stimuli displayed at the same location as the adapting stimuli. Here we show that the adaptation-induced compression of time is specific for translational motion. Adaptation to complex motion, either circular or radial, did not affect perceived duration of subsequently viewed stimuli. Adaptation with multiple patches of translating motion caused compression of duration only when the motion of all patches was in the same direction. These results show that adaptation-induced compression of event-time occurs only for uni-directional translational motion, ruling out the possibility that the neural mechanisms of the adaptation occur at early levels of visual processing.

Although there have been major advances over the last few decades in our understanding of how the human brain processes spatial information, time perception remains very much a mystery. Several models of duration perception exist, but none are completely successful. Basically the models fall into two classes: the “dedicated models”, which assume a specific mechanism for measuring time passage, such as the *clock-counter* model and its several variants^1^,^2^,^3^, and “intrinsic models”, such as the *State-Dependent Network* (SDN) model^4^,^5^,^6^, where the information about event duration is derived from the intrinsic activity of neural populations responding to non-temporal features of the stimulus thus acting as interval timers. With the second class of models, time processing is ubiquitous throughout the brain, rather than being specialized to encode specifically temporal information.

There is a good deal of evidence that event duration can be influenced by many factors, including motion. For example, objects in motion, or contrast-reversed over time, seem to have longer duration than stationary objects^7^,^8^,^9^. Perceived duration is also affected by adaptation: after viewing fast translating stimuli, the perceived duration of a subsequent, slower, stimulus presented in the adapted location is underestimated by about 20%^10^. Importantly, alteration of perceived time is independent of that produced by direct variations of stimulus speed, as it occurs even after compensating speed to offset the effects of adaptation^11^ or adapting to sequences of fast-slow motion that do not affect perceived speed^12^. Adaptation-induced aftereffects on perceived time do not generalize to the entire visual field but are spatially selective to the region that has been adapted^10^,^12^: Burr *et al.*^11^ went on to show that the spatial-selectivity is spatiotopic not retinotopic, suggesting that the reference frame of the adaptation-induced distortions of event time is in real-world, rather than retinal coordinates (but see also refs 13, 14, 15).

It is still far from clear why adaptation to fast-moving stimuli should affect event-time, especially as it does so directly, not via perceived speed or other intermediate mechanisms. A good first-step towards understanding the mechanisms would be to define the conditions under which the compression occurs. Most previous studies have used simple translational or counter-phased motion^10^,^11^,^12^,^13^,^16^,^17^. From the few available studies, it would seem that adaptation to complex motion does not strongly distort duration estimates. For example, Curran and Benton^17^ showed that adaptation to an upward drifting plaid defined by two superimposed sinusoidal gratings produced strong compression of perceived time, of about 10–15%. However, when the two adapting motion components comprised transparent clouds of dots (each individually distinguishable), duration estimates remained veridical. Arrighi and colleagues^18^ reported that adaptation to biological motion had little effect on perceived duration. Both studies suggest that adaptation to complex motion does not affect perceived duration.

To better understand the mechanisms underlying the interactions between visual motion and event time, we investigated in this study whether complex motions, such as expansion or rotation affect perceived duration. For simple and complex motions we measured the effects of adaptation to fast motion on perceived duration, when the speed of the adapted and non-adapted stimuli were perceptually matched. We then investigated whether adaptation to one motion profile distorts temporal estimations of subsequent stimuli moving along different trajectories to test whether and to what extent distortions of perceived time generalized across different kinds of visual motion. Finally, we investigated whether adaptation to motion that entails multiple motion directions (characterizing rotational and radial motion but not linear translation) is a key factor in causing time distortions.

## Results

**Figure 1:**
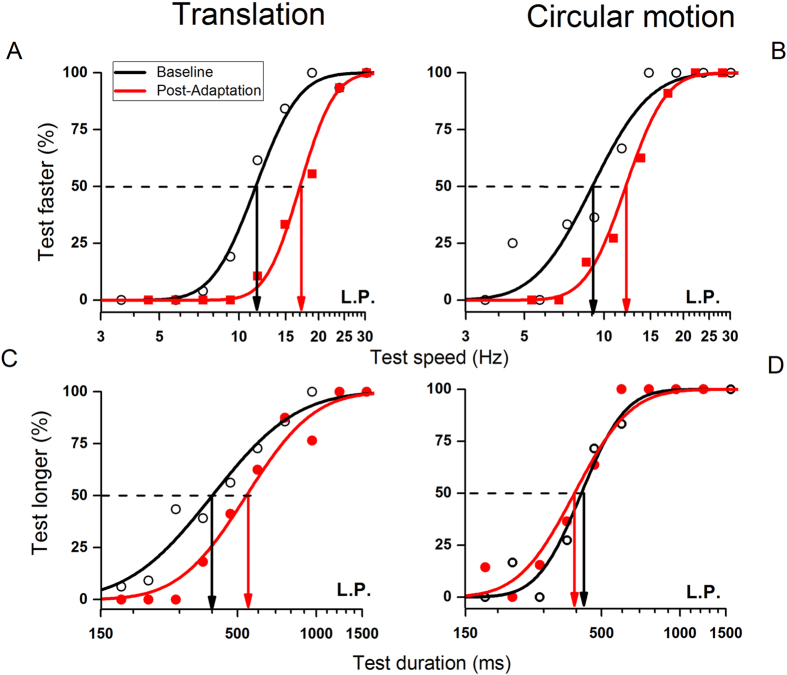
Psychometric functions for the speed or duration discrimination tasks. Psychometric functions of a typical subject showing the proportion of trials the test stimulus appeared to move faster or to last longer than the reference stimulus (fixed duration and speed, 500 ms and 10 Hz respectively), as a function of speed (panels **A,B**) or duration (panels **C,D**) of the test. Data in black refer to the baseline condition in which the discrimination tasks were performed without adaptation. Data in red refer to the adaptation condition in which subjects, after adaptation to fast motion, discriminated either the speed or duration of the test stimulus presented in the adapted location, relative to a reference stimulus presented in a neutral position. The results indicate that adaptation to both motion profiles, translational and circular motion, distorted perceived speed as shown by the rightward shift of the red curves in panels (**A**,**B**). However, apparent duration was compressed just after adaptation to translational motion (rightward shift of the red curve in panel (**C**) but not to circular motion (almost overlapping curves in panel (**D**) when stimuli speed was perceptually matched.

We measured the effect of adaptation to motion on apparent speed and apparent duration (in separate sessions). Figure 1 shows typical psychometric functions – the proportion of trials where the test stimulus (in the adapted region) appeared to move faster or appeared of longer duration than the reference (in an unadapted region) – as a function of speed or duration of the test. Results after adaptation are shown by filled circles, baseline by open circles. For the speed measurements (panels A&B), adapting with both circular and translational motion had similar effects, shifting the red curves rightwards, towards higher speeds (to compensate for the reduction in perceived speed). The point of subjective equality (PSE) is given by the median of the psychometric functions. For both translational and circular motion, in the baseline condition the PSE was near the physical speed of the reference grating (10 Hz), but shifted to around 13–17 Hz after adaptation.

The effect on perceived duration was quite different (panels C&D). Adaptation to translational motion caused a clear change in perceived duration, requiring on average about 30% increase to compensate for the effect of motion-adaptation (which decreased apparent duration), consistent with previous research^11^,^16^. However, adapting to circular motion had no effect on apparent duration: the PSEs for baseline and adapted condition were virtually identical. Note that for the duration judgments, the speed of the test was adjusted to appear identical to the probe, following similar procedures to those used in previous research^11^,^16^.

**Figure 2:**
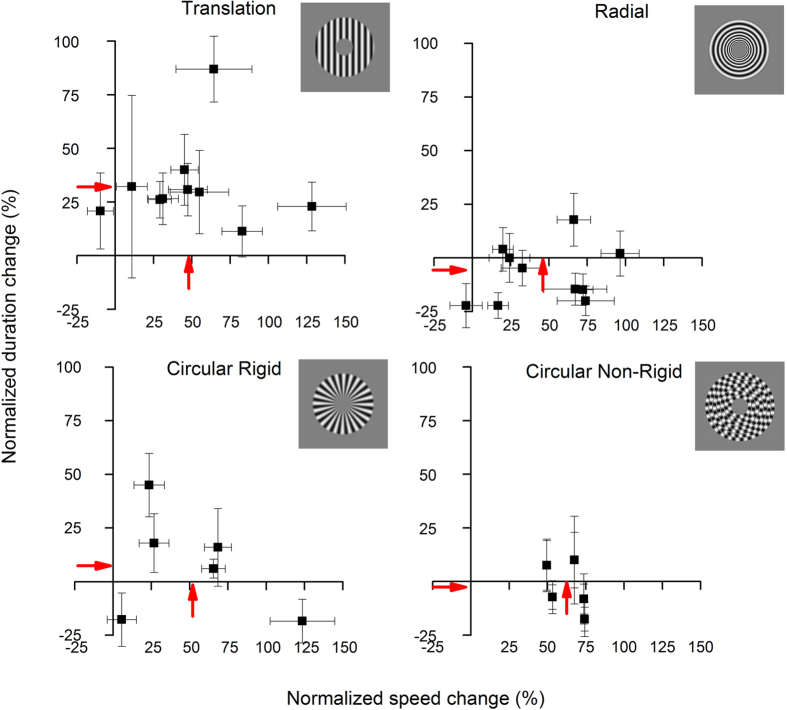
Effects of adaptation to fast motion on perceived speed and duration. Scatter plot showing the effect of motion adaptation to four different kinds of motion on both, perceived speed (abscissa) and duration (ordinate). While adaptation to fast motion affected perceived speed regardless the motion profile taken into account (on average by about 50–60% as shown by the red arrows on the abscissa axes), perceived duration was affected by adaptation only to translational motion (average normalized duration change around 30%). After adaptation to non-translational motion, the duration estimates remained veridical.

Figure 2 shows the effect of motion adaptation on apparent speed and duration, for all subjects for the four different conditions: translation, radial, rigid and non-rigid circular motion. The red arrows near the axes show the group averages. The magnitude of the effect varies considerably between subjects; but while translational motion clearly affects both perceived duration and perceived speed, the other forms of motion affect only speed, not duration, on average. We ran a Multivariate analysis of variance (MANOVA) with changes in perceived speed and duration as the two dependent variables and motion profiles as the independent variable at 4 different levels. The results indicate that there was no significant effect of motion profiles on changes of perceived speed of the adapted stimuli (F_(3,29)_ = 0.265, p = 0.85), but a significant effect of motion profiles on changes in perceived duration (F_(3,29)_ = 11.89, p < 0.001). Post-hoc analysis showed that this difference was due to translational motion being statistically different from radial motion (p < 0.019), circular rigid motion (p < 0.02) and circular non-rigid motion (p = 0.02) with no difference amongst the non-translational motion profiles (all p values > 0.2).

**Figure 3:**
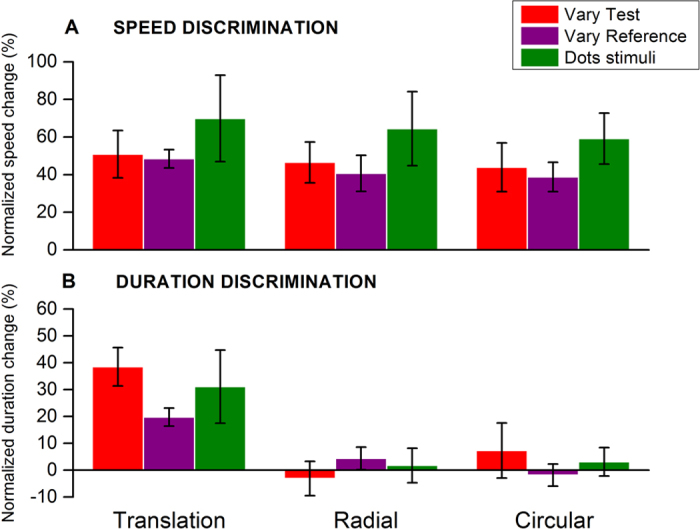
Average effects of adaptation on speed and duration for three different experimental conditions. Upper graphs show the effects of adaptation on perceived speed, for three types of motion, translation, radial and circular while lower graphs show the effect of adaptation on perceived duration. The three experimental conditions are: “Vary test” – results taken from Fig. 2 (except for circular non-rigid condition), where stimuli in the adapted position were varied; “Vary reference” – where the stimulus in the non-adapted position was varied; “Dots stimuli” – experiment with random dots instead of gratings.

In all the experiments discussed above, we measured speed and time PSEs by modulating the stimulus in the adapted position. As this procedure tends to lead to very large compensations in speed, we repeated the experiment (with translational, radial and circular rigid motion stimuli), varying the stimulus in the non-adapted position, keeping constant the adapted one throughout the session to be certain that this procedure did not lead to measurement artefacts. The purple bars of Fig. 3 show the results. Although the effects are slightly smaller, they replicate the findings of the previous experiment: we found a strong reduction in perceived speed after adaptation for all three conditions (baseline versus post-adaptation, p < 0.001, p  <  0.001 and p = 0.002, respectively for translational, radial and circular motion, not different for the three motion profiles: p = 0.420). On the other hand, similarly to the previous experiment, we found a robust effect of perceived duration compression (Fig. 3b) only for translational motion stimuli (baseline versus adaptation, p < 0.001, p = 0.243, p = 0.756), which was significantly different from the effect of the other two motion profiles (translation versus radial, p = 0.026; translation versus circular, p = 0.009).

Another issue of radial and circular rigid stimuli concerns the difference in spatial frequency from the inner to the outer portions of the patches: spatial frequency decreased in inverse proportion to the radius, which may introduce a variation in perceived speed in different portions of the stimuli, and hence create an acceleration or deceleration pattern from the centre to the periphery. To avoid this problem, we also used circular non-rigid motion stimuli, which showed a similar effect. But to be certain, we also repeated the experiment with clouds of dots moving along translational, radial or circular trajectories. Random-dot patterns have a broadband spatial frequency spectrum, which should not interfere with perceived speed or introduce acceleration or deceleration patterns by itself. As shown in Fig. 3 (green bars), we found a very similar pattern of results compared to the previous conditions. While adaptation to these stimuli affected the perceived speed irrespective of the motion profile, the effect on apparent duration was again specific for the translational motion profile (baseline versus post-adaptation comparison significant for all the speed conditions, p-values < 0.002, with no significant difference between different conditions, p-values > 0.05. For duration baseline versus post-adaptation comparison significant only for translation, p = 0.0257; p-values > 0.05 for the other conditions; translational motion effect significantly different from the other conditions, p = 0.044 and p = 0.028, respectively for translation versus radial and versus circular). Overall, these two additional experiments reinforce the main one, showing that the observed pattern of results was not affected by measurement issues.

**Figure 4:**
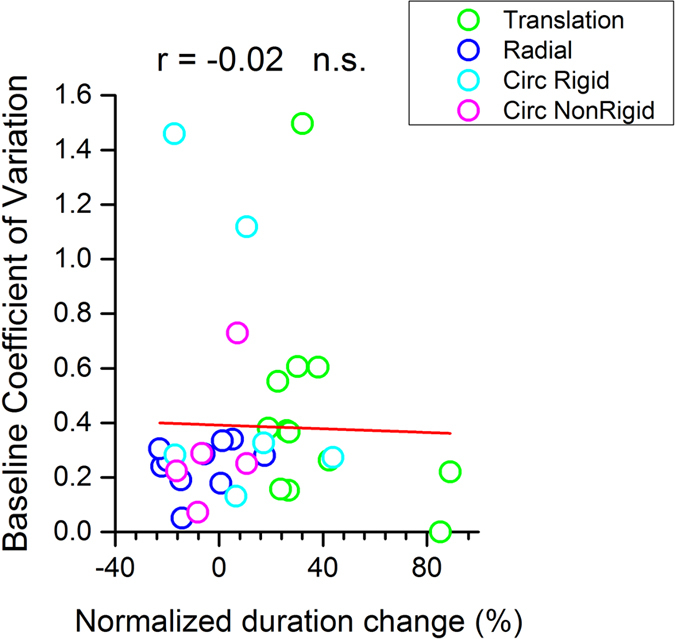
The relation between baseline performances and adaptation effects. The amount of duration change induced by adaptation (abscissa) plotted against the Coefficient of Variation (CoV, defined by thresholds for duration discrimination normalized by the reference duration) before adaptation, for four different motion profiles (data in different colours). Pre-adaptation CoV did not correlate with duration compression, which makes unlikely that the difference in adaptation effects of different kind of motion profiles results from poorer duration discrimination for translational motion.

Why should adaptation affect duration only for translating images? One possibility is that we are less precise at estimating the duration for translating images, and this imprecision leads to a larger adaption effect. For example, Van Der Burg, Alais and Cass^19^ showed a clear correlation between the magnitude of the adaptation effect and size of the simultaneity window. To test this possibility, we calculated the Coefficient of Variation (CoV) for the various tasks, defined as the standard deviation of the psychometric functions divided by the duration of the probe (500 ms). We plot baseline CoV in Fig. 4 as a function of adaptation magnitude (the change in PSE normalized by the pre-adaptation PSE). It is obvious that there is no relationship between the two measures. Pearson’s correlation coefficient r = −0.02, not significantly different from zero. In addition, there was no significant difference in CoV between the four conditions (one-way ANOVA on ranks, H = 4.22, df = 3, p = 0.238). Thus it seems unlikely that the failure to adapt with non-translational motion results from poorer duration discrimination for that type of motion.

**Figure 5:**
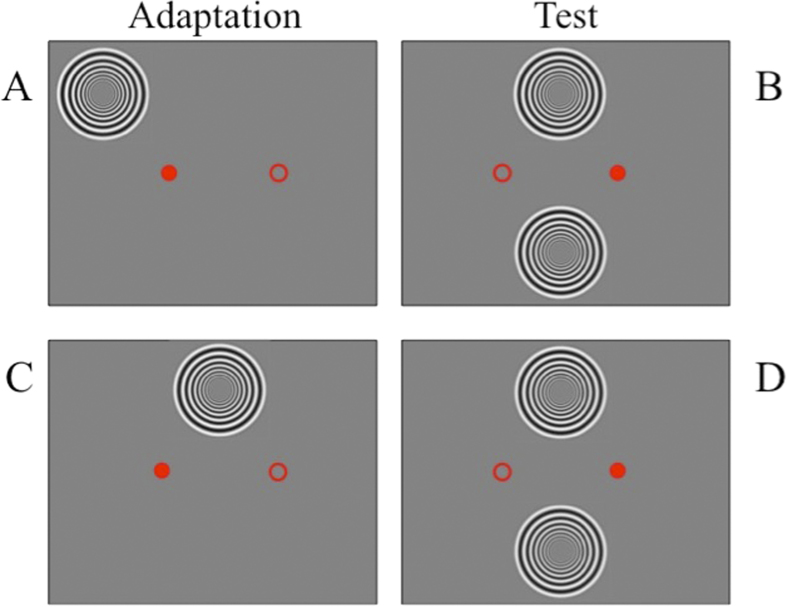
Measuring adaptation after effects in retinotopic and spatiotopic coordinates. A cartoon showing stimuli displacement for the three different experimental conditions. When the adaptation was in retinotopic (eye-centred) coordinates, subjects adapted to a fast moving stimulus displayed on the left of the fixation point, then they executed a rightward saccade and were presented with a test stimulus in the corresponding retinal adapted location (panels **A,B**). In the spatiotopic condition the adaptor was superimposed to the test stimulus after the saccade in external world coordinates but not in the retinal space (panels **C,D**). The full adaptation condition was identical to the spatiotopic condition but with fixation maintained throughout the trial. In all conditions, subjects had to judge either the duration or the speed of the adapted stimulus, relative to a reference displayed in a neutral location below the screen midline.

In previous studies, we have examined whether motion-induced time compression is spatially specific in retinotopic or spatiotopic coordinates: there has been much debate of this issue, with some data suggesting that the effect is primarily spatiotopic^11^,^16^, while others fail to confirm this^13^,^14^. We repeated the measurements here using the method illustrated in Fig. 5. In the test phase of all conditions, subjects viewed a fixation point displayed 6.5° to the right of the screen centre (filled circle in the right-most panels), both test and probe gratings were to the left of the fixation point, the test above the reference below the screen midline. For the retinotopic condition (upper panels), the adaptor was placed to the upper-left of the pre-saccadic fixation point (displayed 6.5° on the left of the screen centre as shown by the filled circle of the left-most panels), in the same retinal position as the test stimulus relative to post-saccadic fixation point. In the spatiotopic condition (lower panels) the adaptor was to the upper right of fixation, in the same screen position as the test after the saccade. In both cases, after adaptation was completed, the fixation point moved to its rightward position and subjects saccaded to it. The test grating appeared 500 ms after extinction of the adaptor, and the reference 500 ms after extinction of the test. The “full” adaptation condition was like the spatiotopic condition, except subjects kept fixation on the left fixation point throughout the experiment.

**Figure 6:**
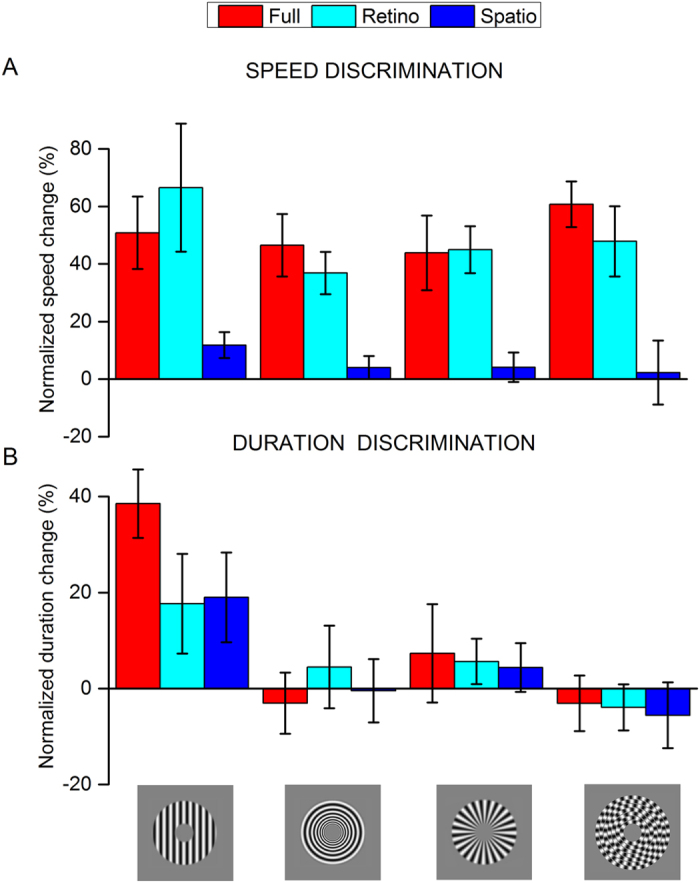
The reference frame of motion adaptation aftereffects on perceived speed and duration. Panel (**A**) Speed change (nornalized by baseline) after adaptation to four different kinds of motion (translational, radial, rigid and non-rigid circular motion), in three different reference frames: retinotopic, spatiotopic or both (full adaptation). In the two conditions with a retinotopic component (retino and full, cyan and red bars respectively), the perceived speed of the adapted stimulus was robustly reduced. On the contrary, speed estimates for stimuli presented in the same spatiotopic location of the adaptor (blue bars in the figure), remained quite veridical with rather no change in the perceived speed of the adapted stimuli. Panel (**B**) Effects of motion adaptation on perceived duration. For translational motion, we found a robust compression of perceived duration for the conditions in which the adaptor and test were superimposed in retinotopic or spatiotopic coordinates, with the sum of these effects to be similar to that measured in the full adaptation condition (compression of about 40%). However, adaptation to radial and circular motion (both versions, rigid and non rigid) did not yield any compression of perceived duration after the speed of the test and reference stimuli was equated. This result indicates that motion adaptation compresses perceived time just after adaptation to translational motion and this effect does not generalize to different kinds of motion profiles.

Figure 6 shows the results for the four types of motion: translation, radial and rigid and non-rigid circular motion. The upper panel shows the results for the effects on apparent speed. All forms of motion caused strong and significant effects in the full condition, around 40% reduction of perceived speed. Also in the retinotopic condition, the reduction in perceived speed occurred for all types of motion, to a similar extent as the full-adaptation condition. However, in the spatiotopic condition, there was no significant change for any of the four types of motion. This result is consistent with the fact that the motion aftereffect is retinotopic, as previously reported^20^,^21^,^22^,^23^.

However, the results for duration compression are quite different. Only adaptation to translational motion yielded a significant compression of perceived duration regardless the reference frame the adaptor and test were superimposed in. As observed before^11^,^16^, the effect was strong in the spatiotopic condition, nearly half the full adaptation condition. There was, however, also an effect in the retinotopic condition, which had not been reported in previous studies of our lab^11^,^16^ but had been observed elsewhere^13^,^14^. For the three types of complex motion, however, none of the conditions caused any compression of perceived duration. This shows that the difference between the effects of adaptation to translational and complex motion on duration perception is very robust.

**Figure 7:**
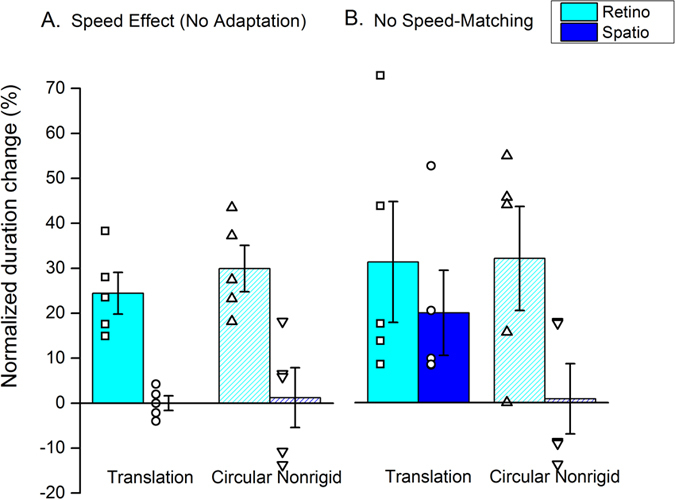
The role of differences in stimuli perceived speed for adaptation-induced distortions on perceived time. Panel (**A**) The effect of the speed difference in estimating stimuli duration. Subjects performed a duration discrimination task between 2 stimuli with the speed of one of them reduced to “simulate” adaptation-induced distortions. The results show that duration estimates for stimuli moving at lower speed were compressed for both kinds of motion, translational and circular non-rigid motion (full and hatched bars respectively). In one condition (data in cyan) we reduced the speed of the test stimulus according to the speed changes found for retinotopic adaptation whilst in the other (data in blue) speed reduction simulated adaptation in the spatiotopic reference frame (data derived from panel (**A**) of Fig. 6). Panel (**B**) Change in perceived duration induced by adaptation when the apparent speed of the adapted and reference stimulus was not equated. For translational motion, duration compression was found in both retinotopic and spatiotopic conditions with the first being strengthened relative to the values found in speed matched conditions (see panel (**B**) of Fig. 6) by the lack of speed compensation given that adaptation did not affect significantly perceived speed in the spatiotopic condition (blue bars in panel (**A**) of Fig. 6). For radial motion, we found a consistent compression of perceived time in retinotopic condition. This indicates that circular motion can affect perceived time, but not by adaptation when adapted and neutral stimulus perceived speed are equated.

Can complex motion influence perceived duration under any circumstance? To test this we measured the effect of varying stimulus speed on perceived duration, using a technique similar to that of Kanai *et al.*^7^. The probe stimulus moved at 10 Hz, while the test moved at a slower speed, calculated to be the same as the speed reduction after adaptation to fast motion (values taken from Fig. 6). The bars of Fig. 7A show that varying image speed had a robust effect on perceived duration, causing a compression of around 25% for both translational and circular motion, as shown in the figure by solid and hatched bars respectively. A two-way RM ANOVA with Holm-Sidak multiple comparisons revealed a statistically significant difference between the duration estimates for the baseline condition (in which stimuli moved at the same physical speed) and the condition in which the test speed was reduced to simulate the effect of adaptation (p < 0.001 for both the motion profiles). The reduction in perceived duration occurred only in the retinotopic condition, because spatiotopic adaptation caused little change in perceived speed in either translational or circular motion (see panel A in Fig. 6), so the test and probe had the same physical speed during the match. These results indicate that circular motion can influence apparent duration; it is only *adaptation* to circular or radial motion that has no effect on duration when adapted and neutral stimulus perceived speed are equated.

The reason for using speeds corresponding to the reduction caused by adaptation was to simulate the effect of making duration judgments after adaptation to fast motion, without compensating for the effects on perceived speed. The bars of Fig. 7B show duration matches for an experiment where perceived speed was NOT compensated for: it was like the experiment of Fig. 6B, except that the test and probe gratings moved as the same physical, rather than apparent speed. Under these conditions, when motion adaptation occurred in retinotopic coordinates, the circular motion had a significant effect on perceived duration similar as that for translational motion (two-way RM ANOVA with Multiple comparison, Holm-Sidak method; baseline versus adaptation, p < 0.05 for both the motion profiles; no statistically significant differences between translational and circular motion, p > 0.05). In both cases, the reduction in perceived duration was similar in magnitude to the effect of varying speed (Fig. 7A). It was therefore probably caused by the same mechanism: the difference in perceived speed, rather than a direct effect of adaptation on duration. Importantly, there was no duration compression in the spatiotopic condition for circular motion (no difference between baseline and adaptation measures, p > 0.05; statistically significant difference between retinotopic and spatiotopic condition, p = 0.036), but only for translational motion (baseline versus adaptation, p = 0.021; no differences between retinotopic and spatiotopic effect: p = 0.420). It has been argued elsewhere^11^ that the spatiotopic effect of duration component is the high-level direct effect of motion-adaptation on duration, not to be confused with the indirect effects via changes in perceived speed.

**Figure 8:**
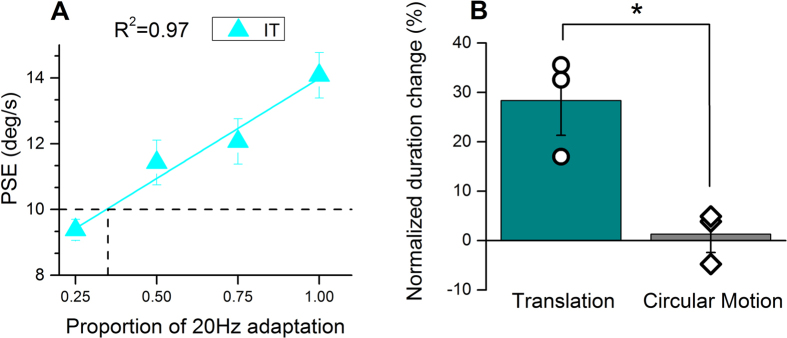
Difference in physical speed between test and reference stimulus cannot account for adaptation effects on perceived time. Panel (**A**) Changes in the perceived speed of the adapted stimulus for different proportions of fast (20 Hz) or slow (5 Hz) adapting speed (data for the typical subject I.T.). The speed ratio that did not change the perceived speed of the adapted stimulus (indicated in the figure by dashed lines) was chosen to measure the effect of adaptation on perceived duration without manipulating the physical speed of the test stimulus. Panel (**B**) Even with this technique, motion adaptation to translational stimuli distorted perceived duration of the test stimulus whilst adaptation to circular motion did not (data averaged across 3 subjects).

The results to date suggest that if the artefact of adaptation-induced speed change is eliminated, there is no adaptation-induced duration compression for complex radial or circular motion. However, if the speed change is not compensated, an effect emerges. Although we compensated for changes in perceived speed in two ways (changing either the reference or the test speed), to be certain that the results are not in some way an artefact of speed compensation, we measured the effect with another technique that causes no changes in apparent speed^12^. We adapted with an interleaved mixture of fast and slow gratings (20 and 5 Hz) of variable ratio: the more high-frequency in the mix, the more it decreases the apparent speed of the test, and vice versa. For each subject we measured the effect on apparent speed as a function of mix ratio (for both translational and circular motion), producing curves like that of Fig. 8A. We then chose the ratio that produced no speed-change for both, translational and circular motion for each subject, and measured the effect on duration for that ratio. The results are shown in Fig. 8B. Again, only for translational motion we found an adaptation-induced compression of perceived duration whilst estimates for circular motion remained rather veridical.

### Adaptor or test?

**Figure 9:**
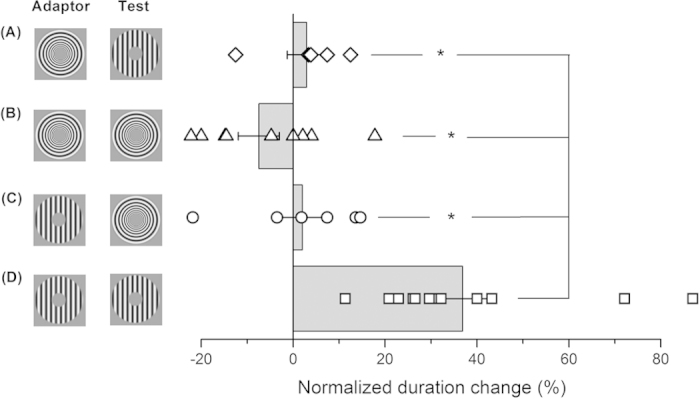
Adapting or testing translational motion? The amount of duration change induced by adaptation to several combinations of adapting and test stimuli. The results indicate that the only condition providing a consistent distortion of perceived time (average compression of about 40%) is the one just comprising translational motion (condition (**D**) in the figure). In all conditions involving radial motion, either as adaptor or as test stimulus, duration estimates remained rather veridical (panels **A,B & C**).

All experiments reported so far consistently show that adaptation to translational motion, but not other types of motion, distorts perceived duration. Is it essential for the adaptor to be translating? Or the test? Or both? To address this issue we tested motion adaptation induced duration compression with 4 different combinations of adaptor and test stimuli: radial-translational; radial-radial; translational-radial; translational-translational (icons in Fig. 9). The bar graphs show that the only combination to cause duration compression was when *both* adaptor and test where translational motion (condition D; Signed Rank test, p < 0.001): if *either* the test or the adaptor were in radial motion, there was no effect as shown by a two-tailed paired t-tests (t = 0.784, p = 0.47, for condition A, t = 1.922, p = 0.086 for condition B, and t = 0.178, p = 0.865 for condition C).

### Decomposing complex motion

**Figure 10:**
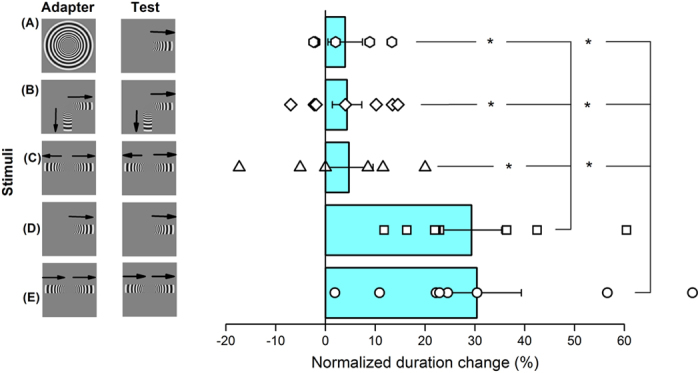
Adaptation to multiple motion directions does not affect perceived duration. Perceived duration change for several combinations of simplified versions of translational and radial motion. Adapting to a full radial patch did not affect duration of a subsequent radial patch in which a single motion direction was left visible (panel **A**). Similarly, when the radial patch was occluded to have two visible sectors either orthogonal (panel **B**) or collinear, vignetting motion in opposite direction (panel **C**), motion adaptation did not compress perceived duration. However, when subjects adapted to a radial patch in which a single sector was visible (panel **D**), the duration of the subsequent stimulus was robustly compressed. The same occurred for the condition in which congruent linear motion was vignetted in two collinear sectors as that shown in panel (**E**). Even in this case we found a strong compression of perceived time to indicate that motion adaptation affect time processing just in those conditions in which motion is defined by a single motion direction.

With translational motion, the local motion is, by definition, all in the same direction and speed. However radial and circular motion comprise motion vectors of all directions, including orthogonal and opposite direction. To test the importance of the different motion directions, we measured duration compression with simplified versions of translational and radial motion, comprising just one or two sectors (icons of Fig. 10). We first adapted with a full radial pattern and tested with a single sector, subtending 1.1 × 4.2° (Fig. 10A). This produced a compression of just about 4%, not significantly different from zero (2-tailed paired t-test: p = 0.26). Similarly, adapting to a simplified radial-motion stimulus defined by 2 motion directions – either orthogonal (Fig. 10B) or opposite (Fig. 10C) – did not yield significant changes in perceived duration of stimuli presented in the adapted location (p = 0.14 and p = 0.38 respectively).

However, adapting to a single motion sector of radial motion, where the local motion was almost in the same direction, did robustly compress the duration, by 29% (p = 0.0015: Fig. 10D). Also adapting and testing to two opposing sectors, both moving in the same direction causes strong duration compression, 30% (p = 0.006). This is clear: what determines whether adaptation causes duration compression is whether the motion is in the same direction over the entire field.

## Discussion

The results of this study clearly show that adapting to fast translational motion reduces perceived duration, by about 30%; while adapting to more complex trajectories, such as radial or circular motion (rigid or non-rigid), or patches of grating with opposing or orthogonal motion, does not affect perceived duration. What seems to be important is that the motion be in a single direction.

Why does adaptation only to single-direction translational motion affect perceived duration? We can exclude that these results arise from poorer precision in duration judgments for translation: there was no significant difference in the discrimination thresholds for the four types of motion used; nor there was a significant correlation between baseline threshold and magnitude of the effect for any of the experimental conditions. If the adaption depended on higher thresholds, one would have expected this correlation to be strong and significant, as has been observed in other conditions, such as adaptation of perceived audio-visual synchrony^19^. Clearly there is something qualitatively different about translational motion that adapts timing mechanisms.

It is important to note that while adaptation to circular motion does not compress duration, circular motion can affect perceived duration directly. The duration of a stimulus rotating at 10 Hz is perceived to be longer than stimuli rotating more slowly. Similarly, if the effect of adaptation to fast rotational motion on apparent duration is measured without compensating for the effect on perceived speed (so the adaptor and test stimuli appeared to be of different speeds), then this will also affect duration. However, when the effect on speed is compensated for directly, or if one uses an adaptation technique that does not change perceived speed^12^, then there is no effect on perceived duration. The results clearly show that while all types of motion have a direct effect on duration, with slower-moving objects appearing of shorter duration (and *vice versa*), only translation causes an adaptation effect.

This result of selective adaptation-induced temporal compression finds broad agreement in the literature. As mentioned in the introduction, Curran and Benton^17^ showed that adaptation to two individually discernable sheets of transparent random-dot motion has little effect on perceived duration, whereas a plaid (also comprising two components) moving in a single direction does. On the other hand, Marinovic & Arnold^24^ have demonstrated adaption-induced duration compression with a rotating pattern of filled circles. It is not obvious why their results were different from ours. Possibly, the main difference was that the circle pattern was quite sparse and the individual elements quite large, so it was not perceived as a single rotating stimulus. It may be interesting to replicate the effects with Marinovic and Arnold’s stimuli to try to understand better the differences. Hogendoorn and coll^25^ reported a change in perceived time after adaptation to radial motion. However, the magnitudes of the effects were small, in the order of 10 ms, compared with 20–40% compression (around 200 ms) observed here and in previous studies^10^,^11^,^13^,^15^. The methods used by Hogendoorn *et al.* were different from those here, it is difficult to make precise comparisons: but certainly the magnitude of the effects reported for adaptation to radial motion are far smaller than those usually reported for adaptation to translational motion and this is perfectly in agreement with the present results.

But why should translational motion, and only translational motion, adapt a specific region of space so that subsequently presented stimuli have reduced duration? This is a difficult question, for which we do not have a complete answer. However, the data of this study help to constrain the explanations for adaptation-induced duration compression. For example, Johnston and colleagues^12^,^26^,^27^ have suggested that duration compression results from preferential adaptation of magnocellular neurons in the lateral geniculate nucleus. Evidence in favour of this idea includes the fact that motion too fast to be processed by cortical units (but presumably not too fast to elicit magnocellular activity in the geniculate) can cause duration compression^28^. However, the fact that circular motion is equally effective in stimulating neurons in the lateral geniculate but it does not cause duration compression, seems to rule out this possibility.

The key difference between stimuli that did and did not adapt duration was whether there was more than one direction of motion displayed in the adapting stimulus (even when the test stimulus was unidirectional). It is far from clear why this should be so. There is evidence that different cortical regions are stimulated by unidirectional and flow motion, possibly corresponding to area MT and MST^29^,^30^,^31^,^32^,^33^. TMS studies have linked activation of the MT/MST complex to time processing^34^,^35^: possibly only the area that responds to unidirectional motion is linked closely with the perception of duration. It may also be of interest that the MT complex receives input directly from sub-cortical structures, bypassing V1. The input comes from the pulvinar^36^, the lateral geniculate nucleus^37^ and the superior colliculus^38^. It is possible that this direct pathway is differently stimulated by uni- and multi-directional motion, leading to different adaptation effects.

It is still far from clear what functional role motion-induced compression of event duration may serve. We know that the adaptation is spatially specific^10^, and many studies suggest that the spatial specificity may be largely spatiotopic (although the current results, as well as much research from other laboratories^10^,^13^,^14^, suggest there may also be a retinotopic component). The spatiotopic component in the selectivity points to relatively high-level processing areas. However, it remains far from clear why adaptation to motion affects the timing properties of these areas; and far less clear why the adaptation should need to be unidirectional.

We are fully aware that the above discussion is speculative. However, while the interpretation may be speculative, the facts are very clear: adaptation to fast translational motion causes robust changes to perceived duration, while adaptation to circular or radial motion, or to two sectors of motion in opposing or orthogonal directions does not. We believe that understanding this phenomenon will be important in understanding fully the mechanisms underlying event duration, and their interconnection with the dynamic perception of space.

## Methods

### Subjects

A total of 25 subjects participated in the several experiments of the study (mean age 25 years), with some participating in either a single or multiple experimental conditions. All participants had normal or corrected-to-normal visual acuity, gave informed written consent and were naïve to the purpose of the study (with the exception of Author M.F. who participated in all experiments). The study was approved by the local research ethics committee (Azienda Ospedaliero-Universitaria Pisana n. 45060) and adhered to the Declaration of Helsinki. In Experiment 1 we tested 12, 10, 6 and 5 participants for translation, radial, rigid and non-rigid circular motion condition respectively. In the experiment in which we manipulated the stimulus presented in the un-adapted location we tested 6 subjects in all conditions whilst 5 subjects participated to the experiment in which the moving stimuli were cloud of dots and not gratings. In the condition in which we tested the reference frame of the adaptation aftereffects (either retinotopic or spatiotopic) we tested 6 participants for the translation, radial and circular motion condition, while 5 subjects were tested in the circular non-rigid condition. In the two experiments in which we investigated the effects of perceived speed on stimuli duration we tested 5 participants in both experimental conditions: “No adaptation” and “No speed-matching”. We tested 3 participants in the experiment in which the speed of the adapting stimulus was adjusted to do not trigger changes in the perceived speed of the adapted stimulus, whilst 6 subjects participated to the experiment in which we tested different combinations of motion profiles for the adapting and test stimuli. Finally, in the final experiment in which we manipulated the visible area of the adapting stimulus we tested 8 participants with the only exception of the “full-patch” adapter condition in which we tested 5 participants. All the group sizes reported above include the only non-naïve subjects M.F. (author of the study) who participated in all the experimental conditions.

### Stimuli and procedures

Visual stimuli were presented on a Barco CRT monitor (Barco Calibrator, screen resolution 1024 × 768, 32 bit colour depth, refresh rate 100 Hz and mean luminance 27 cd/m^2^) subtending about 40° × 30° at subjects viewing distance of 57 cm. Stimuli were generated with the Psychophysics Toolbox V.3^39^ for MatLab (version 2010b) running on a PC computer. All experiments comprised a test phase consisting of the presentation of a sequence of two stimuli, test (variable duration) and reference (fixed duration) with subjects required to indicate which stimulus, test or reference, lasted longer (guessing whenever uncertain). On each trial the duration of the reference stimulus was constant at 500 ms, while that of the test was variable, determined by the adaptive QUEST routine^40^. While in the first trial of the session (usually 30 trials), the test duration was initially 500 ms (same as reference), in subsequent trials, the test duration was the PSE estimated by the QUEST routine, plus a random value drawn from a Gaussian distribution with standard deviation 30 ms. This procedure ensured that there was considerable scatter around the PSE, and that the number of “longer than” and “shorter than” trials were roughly equal. Usually 3–5 sessions were run for each condition, in randomized order. The final estimate of PSE was taken as the median of the best-fitting cumulative Gaussian function to all the data of a particular condition (percentage “longer than” against test physical duration). Examples of these functions are shown in Fig. 1 (lower panels) for duration estimates of two different kinds of motion profiles (translational and radial motion). On separate sessions, subjects performed duration discrimination after being adapted to fast motion. Each trial started with a moving stimulus subtending an area of 5° × 5° displayed for 7 seconds on given location of the visual field. On the following test phase, the test stimulus was presented in the adapted location and the reference in a neutral position at an equivalent distance from fixation. The total amount of adaptation-induced distortions on perceived duration was defined as the difference between the PSEs of the baseline and the adaptation condition (both conditions show in Fig. 1 with black curves and symbols referring to the no-adaptation condition and data in red to the adaptation condition). The total change of perceived duration induced by adaptation was measured as follows:


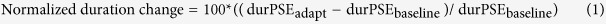


with durPSE_baseline_ indicating the PSE for duration in the baseline condition and durPSE_adapt_ the PSE in the adaptation condition. In most experiments we manipulated the physical duration of the stimulus presented in the adapted location (test stimulus), so positive values of duration change correspond to a perceptual compression of time. However, for consistency, in the conditions in which we manipulated the duration of the stimulus presented in the neutral location (i.e. those reported by purple bars in Fig. 3), we defined changes in perceived duration by the same formula but reversed the sign of the values so we could compare them directly with the others: positive values means compression.

As well as measuring the effect on duration, we measured the effect of adaptation on perceived speed, using a similar procedure. Again one region was adapted to fast motion, and then test and reference were presented as before. Again the reference was constant (at 10 Hz) and the QUEST procedure determined the speed of the adapted stimulus to home in on the speed match. PSE was given as the mean of the psychometric functions, with a minimum of 60 data points (upper panels in Fig. 1).

In most duration discrimination experiments, the physical speed of the test (adapted) stimulus was increased to perceptually match that of the non-adapted reference. We measured for each subject, in each condition, the changes of the physical speed of the test stimulus needed to match the reference speed and we used this value of physical speed in the duration discrimination experiments requiring speed matching. In the graph reporting the effect of adaptation on perceived speed, the overall speed changes were measured similarly as for perceived duration, that is, as the difference between PSE in the adapted condition and baseline condition normalized by baseline following the formula:


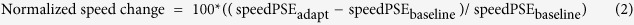


with speedPSE_baseline_ indicating the PSE in the baseline condition and speedPSE_adapt_ the PSE for the adaptation condition. We also used a condition where the stimulus in the adapted position was kept constant (TF 10 Hz, duration 500 ms, as the reference stimulus), while the stimulus in the non-adapted position was varied according to the quest routine. Again, in this condition, the sign of the values was reversed to make easy the comparison with the other results: positive values means reduction of the perceived speed of the adapted stimulus.

We studied adaptation-induced distortions on perceived duration for four different kinds of motion profiles: a) linear translation; b) radial motion; c) rigid circular motion and d) non-rigid circular motion. Translating stimuli were vertical luminance modulated gratings (SF 1 cpd) drifting horizontally, changing drift direction every 2 seconds. Radially moving stimuli were concentric circular gratings with a spatial frequency ranging from 0.5 to 1.2 cpd (outer and inner border respectively), alternatively expanding and contracting, again changing direction every 2 second. Circular motion was tested with both rigid and non-rigid rotation. Rigid-motion stimuli were windmill-like rotating gratings (spatial frequency increasing from 0.5 to 1.2 cpd from the outer to the inner border), while non-rigid rotational stimuli were defined by a series of 7 concentric circle gratings, each of them with a spatial frequency of about 1 cpd. All stimuli were windowed within an annular mask (inner and outer diameters equal to 2° and 10° respectively), with borders blurred by a Gaussian smoothing (spatial constant equal to 0.15 deg) and were presented with a Michelson contrast of 90%.

In a separate condition, we studied translational, radial and circular motion profiles using random-dot patterns. These stimuli comprised 100 dots (50 black and 50 white), each of diameter of 0.15 deg, randomly positioned within a circular area of radius 5 deg. Dots moved along translational (left/right), radial (inward/outward) or circular trajectories, with the speed of each element kept constant at all eccentricities. Each dot had a limited lifetime of 5 frames, after which its position was reassigned randomly to another portion of the stimulus.

In one experimental condition we used an adaptation technique devised by Ayhan, Bruno, Nishida and Johnston^12^, adapting with gratings alternating in speed between 5 and 20 Hz, and varying the ratio of the two speeds to determine a stimulus that would not cause any change in apparent speed (see panel A in Fig. 8). This ratio was then used as the adaptor, without the need to change the speed of stimuli in the adapted location.

In most conditions we measured adaptation-induced distortions on perceived duration with similar motion profiles of adaptor and test/reference stimuli. However, to test whether adaptation to a motion profile affects duration estimates for stimuli moving along different trajectories, we added two additional adaptation conditions in which subjects adapted to translational/radial motion, and subsequently estimated duration of a radial/translational stimulus. Finally, we also performed a series of experiments to investigate the role of multiple motion directions to assess whether this is the key factor determining whether motion adaptation affect perceived time.

## Additional Information

**How to cite this article**: Fornaciai, M. *et al.* Adaptation-Induced Compression of Event Time Occurs Only for Translational Motion. *Sci. Rep.* **6**, 23341; doi: 10.1038/srep23341 (2016).
